# Lung adenocarcinoma harboring concomitant *SPTBN1-AL*K fusion, c-Met overexpression, and *HER-2* amplification with inherent resistance to crizotinib, chemotherapy, and radiotherapy

**DOI:** 10.1186/s13045-016-0296-8

**Published:** 2016-08-05

**Authors:** Fei-fei Gu, Yong Zhang, Yang-yang Liu, Xiao-hua Hong, Jin-yan Liang, Fan Tong, Jing-song Yang, Li Liu

**Affiliations:** 1 Cancer Center, Union Hospital, Tongji Medical College, Huazhong University of Science and Technology, Wuhan, China; 2 Department of Radiation Oncology, Hubei Cancer Hospital, Wuhan University, Wuhan, China

**Keywords:** NSCLC, Lung adenocarcinoma, Oncogenic drivers, *SPTBN1-ALK*, c-Met, *HER-2*, Crizotinib, Chemotherapy, Radiotherapy

## Abstract

Crizotinib is a multi-targeted tyrosine kinase inhibitor (TKI) with activity against mesenchymal-epithelial transition factor (MET) and anaplastic lymphoma kinase (ALK). However, the concomitant oncogenic drivers may affect the sensitivity of crizotinib. Herein, we present a 69-year-old never-smoker Chinese male with advanced lung adenocarcinoma harboring concomitant spectrin beta non-erythrocytic 1 (*SPTBN1*)*-AL*K fusion, c-Met overexpression, and human epidermal growth factor receptor-2 (*HER-2*) amplification with inherent resistance to crizotinib, chemotherapy, and radiotherapy. Although the patient received timely and comprehensive treatment, the overall survival was only 8 months. Therefore, c-Met overexpression, *HER-2* gene amplification, and *SPTBN1*-*AL*K gene fusion can coexist in lung adenocarcinoma and may become a potential biomarker of cancer refractory to crizotinib, chemotherapy, and radiotherapy as well as of a relatively poor prognosis. In addition, the novel *SPTBN1-AL*K fusion gene may become a potential target for anti-tumor therapy.

**Electronic supplementary material:**

The online version of this article (doi:10.1186/s13045-016-0296-8) contains supplementary material, which is available to authorized users.

## To the editor

Crizotinib is a multi-targeted tyrosine kinase inhibitor (TKI) with activity against mesenchymal-epithelial transition factor (MET) and anaplastic lymphoma kinase (ALK) [1]. Driver oncogenes are conventionally considered mutually exclusive [2]. Here, we describe a rare case of lung adenocarcinoma harboring concomitant spectrin beta non-erythrocytic 1 (*SPTBN1*)*-AL*K fusion, c-Met overexpression, and human epidermal growth factor receptor-2 (*HER-2*) amplification with inherent resistance to crizotinib, chemotherapy, and radiotherapy.

**Fig. 1:**
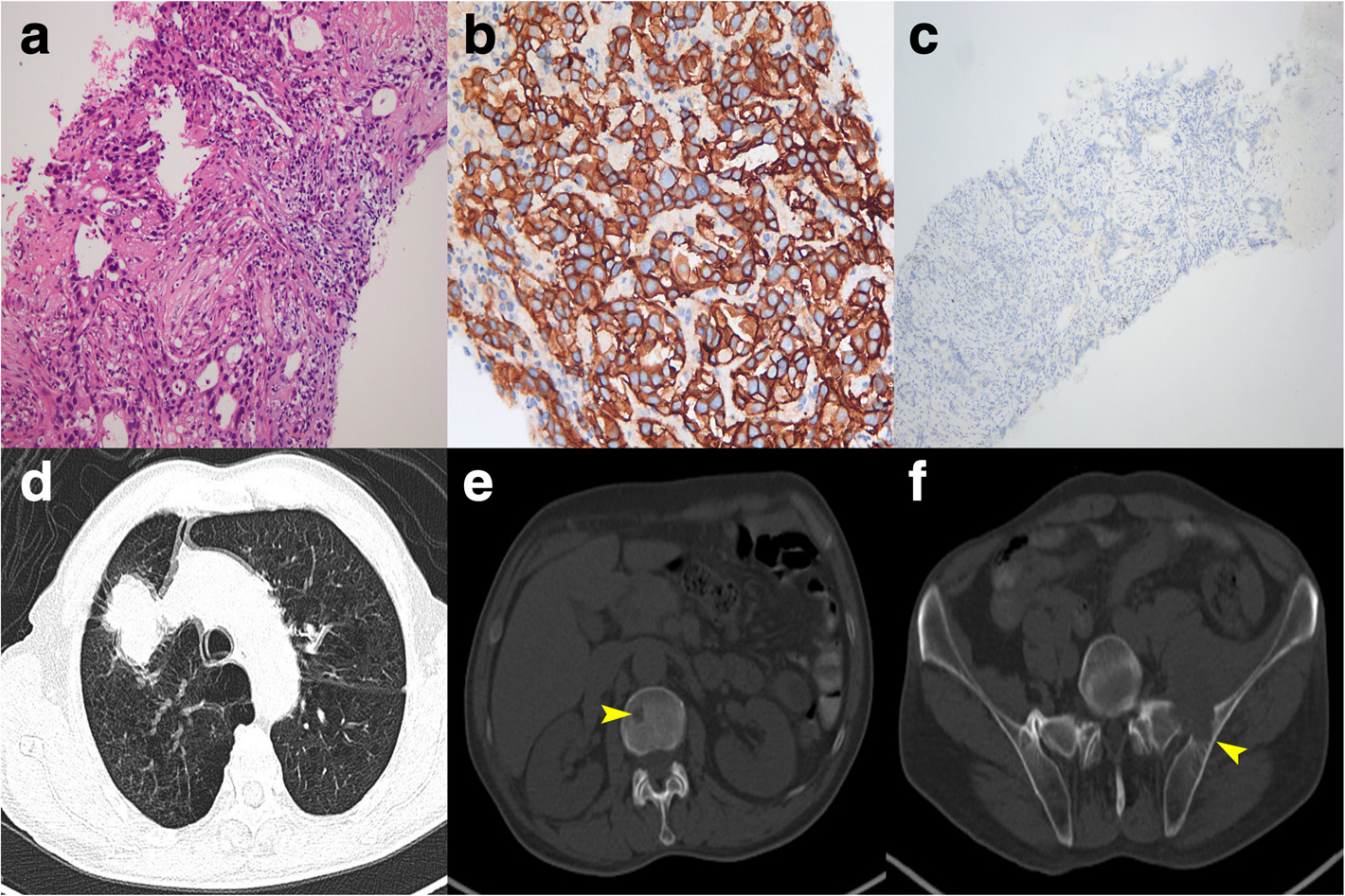
Lung biopsy specimens and total-body PET/CT scan before treatment. Hematoxylin and eosin (H & E) staining (magnification ×200, **a**), IHC of c-Met (magnification ×200, **b**), and ALK (magnification ×100, **c**) of a diagnostic biopsy specimen. Total-body PET/CT showed a 5.1 × 3.7 cm-sized tumor in the right upper lobe (**d**) with the second lumbar and left ilium metastases (*arrowheads*, **e**, **f**)

In July 2015, a 69-year-old never-smoker man, whose brother died of lung cancer, experiencing pain in his low back and left lower extremity, underwent a total-body positron emission tomography-computed tomography (PET/CT) scan which showed a lung tumor and bone metastases (Fig. 1d–f). Then, a CT-guided percutaneous lung biopsy was performed (Fig. 1a), and a diagnosis of advanced lung adenocarcinoma was made. Based on the immunohistochemistry analysis, the tumor cells were negative for ALK (Fig. 1c) but extremely positive for c-Met (Fig. 1b). In addition, epidermal growth factor receptor (EGFR) mutation and repressor of silencing 1 (*ROS-1*) fusion detections revealed negative results.

The patient initially received gemcitabine plus nedaplatin for one cycle in July 2015. Subsequent treatment included palliative radiation therapy to the left ilium. Additionally, the patient was treated with crizotinib based on c-Met overexpression. However, grade 3 thrombocytopenia occurred after chemotherapy, and he recovered with recombinant human thrombopoietin. Although the symptoms decreased, first restaging CT scans (September 2, 2015) showed marked worsening disease (Additional file 1: Figure S1a).

Then, the patient received two cycles of bevacizumab-based chemotherapy with pemetrexed, cisplatin, and bevacizumab from September to October in 2015. Additionally, he received local radiotherapy at the lumbar metastases. However, lumbar MRI and CT scan of the chest and abdomen (October 2015) showed progressive disease (Additional file 1: Figure S1b).

**Fig. 2:**
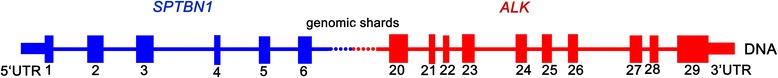
Hypothetical structure of *SPTBN1-ALK* fusion gene. *SPTBN1-ALK* fusion gene was formed by placing *SPTBN1* exons 1–6 upstream of *ALK* exons 20–29, which were separated by small genomic shards

Given the patient’s reduced performance status, reduced paclitaxel liposome plus nedaplatin was administered (October 27, 2015). Subsequent treatments included iAPA DC-CIK and chest radiotherapy. However, the patient demonstrated evidence of progressive disease again in December 2015 (Additional file 1: Figure S1c). To re-evaluate the molecular characteristics, DNA extracted from the original biopsy was used for DNA sequencing with next-generation sequencing on December 2015. Interestingly, the patient had *HER*-2 amplification and a novel *ALK* rearrangement, namely *SPTBN1-ALK* fusion, which was created by an insertion between two breakpoints in exons 1 to 6 of the *SPTBN1* gene and exons 20 to 29 of the *ALK* gene (Fig. 2). Given the patient’s performance interfered with starting chemotherapy, he was treated with whole-brain irradiation therapy. However, the patient’s performance status continuously deteriorated. On January 4, 2016, the patient died of brain and lung metastases. The patient’s overall survival was only 8 months.

The *SPTBN1-ALK* fusion gene was first identified in colorectal cancer, which was formed by the fusion of exon 7 of the *SPTBN1* gene with exon 20 of the *ALK* gene [3]. In this case, we first identified a novel *SPTBN1-ALK* fusion in lung cancer. The frequency of c-Met overexpression is 31.9 % in NSCLC, and it potentially causes intrinsic resistance to EGFR-TKIs without causing resistance to crizotinib [4]. Nonetheless, the present patient gained inherent crizotinib resistance despite harboring both c-Met overexpression and the *SPTBN1-ALK* fusion gene. HER-2 is noted in 10 to 20 % of NSCLC patients [5] and confers relative resistance to conventional chemotherapy [6]. The novel *ALK* rearrangement and interactive crosstalk between Met and HER2 may have been responsible for the failed response to crizotinib treatment. In this context, inhibition of ALK, Met, and Her-2 was required for efficient inhibition of tumor growth.

To the best of our knowledge, this is the first case of lung cancer with a novel *SPTBN1-ALK* fusion gene, which may become a potential target for anti-tumor therapy. This interesting case demonstrates that c-Met overexpression, *HER*-2 gene amplification, and *SPTBN1*-*AL*K gene fusion can coexist in lung adenocarcinoma, and their combination might be a biomarker for resistance to crizotinib, traditional chemotherapy, and radiotherapy as well as for a relatively poor prognosis. Further evidence is required to validate these preliminary data.

## Abbreviations

ALK, anaplastic lymphoma kinase; CIK, cytokine-induced killers; DC, dendritic cell; EGFR, epidermal growth factor receptor; *HER-2*, human epidermal growth factor receptor-2; iAPA, inhibition of antigen presentation attenuators; MET, mesenchymal-epithelial transition factor; MRI, magnetic resonance imaging; NSCLC, non-small-cell lung cancer; PET/CT, positron emission tomography-computed tomography; ROS1, repressor of silencing 1; *SPTBN1*, spectrin beta non-erythrocytic 1; TKI, tyrosine kinase inhibitor

## Additional file

Additional file 1: Figure S1. Multiple metastases after three treatment approaches (a) CT scans of the chest and abdomen showed mediastinal lymph nodes metastases (left, arrowhead), a soft tissue tumor located at the axillary segment of the right sixth rib with bone destruction (middle, arrowhead) and left adrenal metastasis (right, arrowhead) following the first-line treatment. (b) After the second-line treatment, CT scans of the chest and abdomen showed a soft tissue tumor at the right scapula with bone destruction (left, arrowhead) and bilateral adrenal metastases (right, arrowheads). Lumbar MRI showed the third lumbar metastasis (middle, arrowhead). (c) After the third-line treatment, chest CT scans showed multiple bilateral pulmonary nodules and axillary lymph nodes metastases (upper, arrowheads) and brain MRI showed multiple brain metastases (lower, arrowheads). (TIF 13959 kb)
