# Supplementary figures and images for: Lung adenocarcinoma harboring concomitant SPTBN1-ALK fusion, c-Met overexpression, and HER-2 amplification with inherent resistance to crizotinib, chemotherapy, and radiotherapy
Source: J Hematol Oncol. 2016 Aug 5;9:66. doi: 10.1186/s13045-016-0296-8 (PMC4974806; doi:10.1186/s13045-016-0296-8)

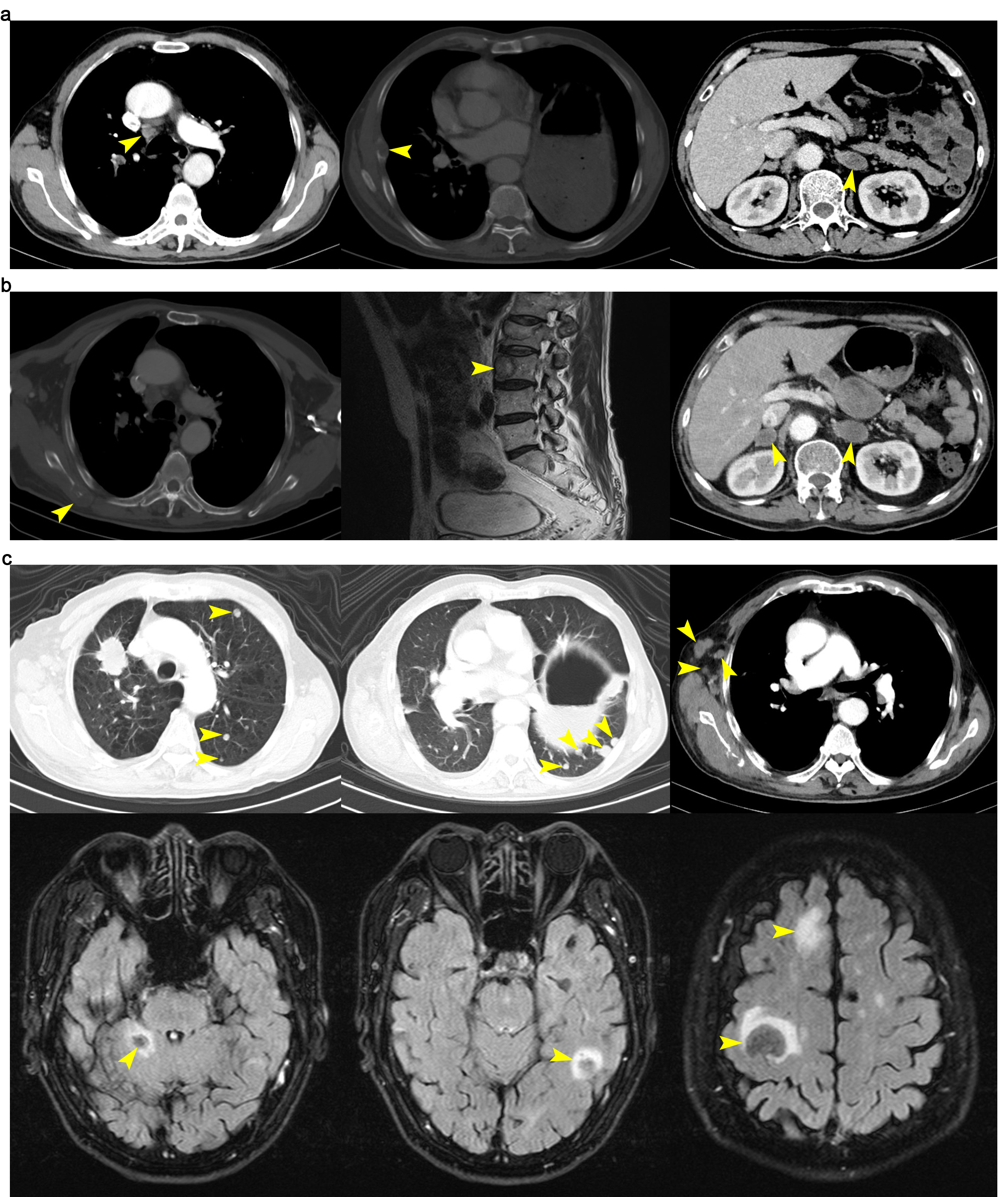

Supplement: Additional file 1: Figure S1. — Multiple metastases after three treatment approaches (a) CT scans of the chest and abdomen showed mediastinal lymph nodes metastases (left, arrowhead), a soft tissue tumor located at the axillary segment of the right sixth rib with bone destruction (middle, arrowhead) and left adrenal metastasis (right, arrowhead) following the first-line treatment. (b) After the second-line treatment, CT scans of the chest and abdomen showed a soft tissue tumor at the right scapula with bone destruction (left, arrowhead) and bilateral adrenal metastases (right, arrowheads). Lumbar MRI showed the third lumbar metastasis (middle, arrowhead). (c) After the third-line treatment, chest CT scans showed multiple bilateral pulmonary nodules and axillary lymph nodes metastases (upper, arrowheads) and brain MRI showed multiple brain metastases (lower, arrowheads). (TIF 13959 kb) [file 13045_2016_296_MOESM1_ESM.tif]
